# Global Impact of COVID-19 Pandemic on Gastrointestinal Infections: A Scoping Review

**DOI:** 10.1089/fpd.2024.0047

**Published:** 2024-11-26

**Authors:** Afroditi Lazarakou, Lapo Mughini-Gras, Roan Pijnacker

**Affiliations:** ^1^ Centre for Infectious Disease Control, National Institute for Public Health and the Environment (RIVM), Bilthoven, The Netherlands.; ^2^ Institute for Risk Assessment Sciences, Utrecht University, Utrecht, The Netherlands.

**Keywords:** COVID-19, nonpharmaceutical interventions, gastrointestinal pathogens, impact, incidence, surveillance

## Abstract

During the COVID-19 pandemic, nonpharmaceutical public health interventions (NPIs) were implemented worldwide to control the spread of severe acute respiratory syndrome coronavirus 2. However, the incidence of other pathogens, including gastrointestinal (GI) pathogens, was also affected. Here, we reviewed studies assessing the impact of NPIs during the COVID-19 pandemic on the incidence of GI infections, particularly foodborne infections. A systems literature search was conducted in May 2023, using Living Evidence on COVID-19 (COAP) and Scopus. Articles were identified and selected through a screening process with inclusion and exclusion criteria based on the Preferred Reporting Items for Systematic Reviews and Meta-Analysis statement. Data were extracted from each full-text article included in the review. Parameters included were GI viruses, GI bacteria, NPIs against the COVID-19 pandemic, and the associated impact of NPIs on GI pathogens. A total of 42 articles were included in the review, representing 18 countries. Overall, a larger reduction was observed for viral GI infections compared with bacterial GI infections during the COVID-19 pandemic, particularly for norovirus. For bacterial GI infections, *Campylobacter* and nontyphoidal *Salmonella* were the most frequently detected pathogens in the majority of the studies, with the largest reduction observed for *Shigella* and Shiga toxin–producing *Escherichia coli* infections. The sharp decrease in GI viral infections in most of the included countries is suggested to be related to the disruption of person-to-person transmission due to several implemented interventions (e.g., social distancing and hand hygiene). GI bacterial pathogens, more commonly transmitted via the foodborne route, were least impacted, and their reduction is associated with closure of food-providing settings and travel restrictions. However, the observed changes appear to be multifactorial; alterations in health-care-seeking behaviors and in routinary diagnostic testing have undeniably played a significant role, affecting national surveillance systems. Therefore, although NPIs likely had a substantial impact on the burden of GI infectious diseases, the extent of the true change cannot be fully assessed.

## Introduction

Since the beginning of 2020, public health authorities and healthcare providers called to control the coronavirus disease 2019 (COVID-19) pandemic, caused by the severe acute respiratory syndrome coronavirus 2 (SARS-CoV-2). In the early stages of the epidemic, when no vaccines were available yet, several nonpharmaceutical public health interventions (NPIs) were implemented, such as social distancing, mask mandates, travel restrictions, school and restaurant closures, as well as quarantine measures to control the spread of SARS-CoV-2. Although the target of such NPIs was SARS-CoV-2, changes in the transmission of other pathogens, including those causing mainly respiratory, sexually transmitted, and gastrointestinal (GI) infections, were also observed (Middeldorp et al., 2021; Mughini-Gras et al., 2021; Soo et al., 2020; Ullrich et al., 2021). Moreover, different prevention and control measures were applied at national and regional levels in different countries, depending on the specific evolution of the epidemic (Antunes et al., 2020).

Commonly applied control measures (e.g., handwashing, social distancing, travel restrictions) can also prevent the transmission of GI pathogens (Palmer et al., 2022). Therefore, it has been suggested that some of the measures implemented during the COVID-19 pandemic could explain the observed reduction in GI pathogen transmission as well. A significant decrease in most notifiable diseases, including those caused by GI pathogens, during the COVID-19 pandemic has been well documented in Germany (Ullrich et al., 2021), with similar results being reported in China (Chen et al., 2021), the United States of America (USA) (Ray et al., 2021), and other countries, mainly across Europe.

Transmission of GI pathogens typically follows the fecal−oral route, with consumption of contaminated food or water being often the main transmission pathway, as well as contact with infected individuals, animals, or the environment (Love et al., 2022). Among the main GI bacterial pathogens responsible for GI infections are *Campylobacter*, *Salmonella*, *Listeria monocytogenes*, and Shiga toxin–producing *Escherichia coli* (STEC), which are mostly associated with foodborne tranmission (Russini et al., 2022), as well as *Shigella*, which is mainly transmitted directly from person to person and indirectly through contaminated food and water in developed countries (Bassal et al., 2021). The main viral agents of gastroenteritis include noroviruses and rotaviruses, which are also mainly transmitted from person to person, but have a foodborne route as well (Nachamkin et al., 2021; Russini et al., 2022). Understanding the extent to which the measures against SARS-CoV-2 also affected GI infections might significantly contribute to the identification and implementation of prevention strategies to control these pathogens. Moreover, different countries might have adopted different approaches in different periods to control SARS-CoV-2 spread; thus, differences in the potential effects of these measures can be expected.

This scoping review aims to synthesize current evidence that measures implemented against SARS-CoV-2 worldwide had also a significant effect on the incidence of GI infections, particularly foodborne infections.

## Methods

This review followed the Preferred Reporting Items for Systematic Reviews and Meta-Analysis statement. This set of guidelines applied.

### Literature search

A systematic literature search was conducted in May 2023 using two online search databases: Living Evidence on COVID-19 (COAP) which includes research about SARS-CoV-2 and COVID-19 up until February 28, 2022, and Scopus. The databases were searched using the following keywords in Title/Abstract: ‘‘covid-19 measures,” “covid-19 restrictions,” “covid-19 lockdown,” “COVID-19 pandemic,” “gastrointestinal infectious diseases,” “gastrointestinal infections,” “gastrointestinal pathogens,” “foodborne diseases,” “foodborne infections,” “foodborne pathogens,” “*Campylobacter*”, “*Salmonella*,” ‘‘STEC’’, “*Listeria*,” “*Shigella*,” “Norovirus,” and “Rotavirus.” A manual search was also performed using Scopus for identifying additional articles, aimed to support the choice and selection of the presented articles (Table 1).

**Table 1.** Search Strategy for Online Search Databases

Search Database	Search Terms Used
COAP, Advanced search	(foodborne diseases) OR (foodborne pathogens) OR (foodborne infections) OR (foodborne infectious diseases) OR (*Campylobacter*) OR (*Salmonella*) OR (STEC) OR (*Listeria*) OR (*Shigella*) OR (rotavirus) OR (norovirus) AND ((COVID-19 measures) OR (COVID-19 restrictions) OR (COVID-19 lockdown) OR (COVID-19 pandemic))
Scopus, Advanced search	(TITLE-ABS-KEY (covid-19* OR “COVID-19 measures” OR “COVID-19 lockdown” OR “COVID-19 interventions” OR “COVID-19 restrictions “OR “COVID-19 pandemic”) AND TITLE-ABS-KEY (“gastrointestinal infectious diseases” OR “gastrointestinal infections” OR “gastrointestinal pathogens” OR “foodborne diseases” OR “foodborne pathogens” OR campylobacter OR salmonella* OR “*Listeria* monocytogenes” OR “STEC” OR norovirus OR rotavirus)) AND PUBYEAR >2019 AND PUBYEAR <2023
Scopus, Manual search	Incidence of notifiable infectious diseases and COVID-19

### Selection process

**FIG. 1.**
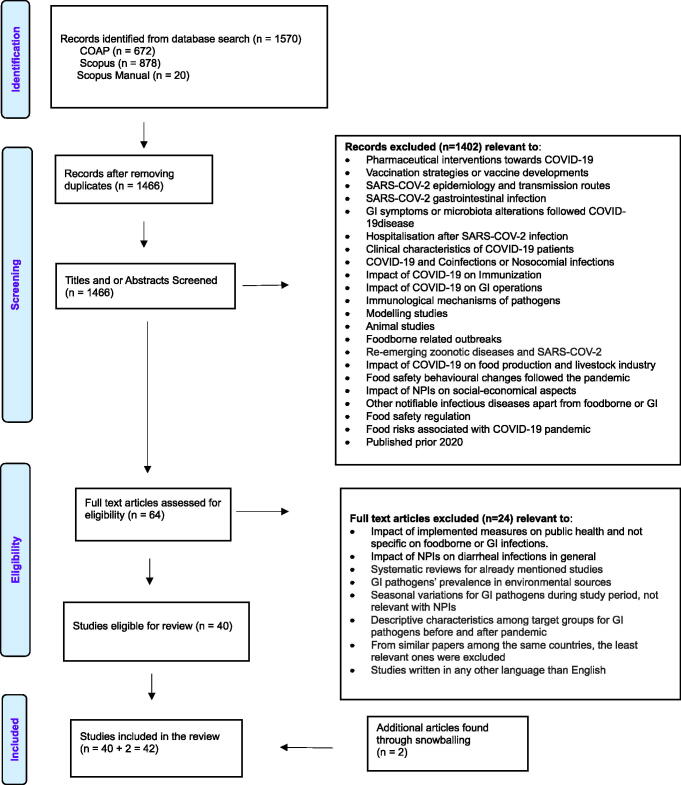
PRISMA flow diagram for study selection process. PRISMA, Preferred Reporting Items for Systematic Reviews and Meta-Analysis.

Selection of articles was done independently by two researchers who screened titles, abstracts, and finally full-text articles using the following inclusion criteria: (1) studies referred to NPIs against SARS-CoV-2 and their impact on foodborne diseases, foodborne pathogens, GI diseases, or GI pathogens in general; (2) studies referred to the specific foodborne and GI pathogens mentioned in the used search terms; (3) studies reported on the incidence or case counts of foodborne and/or GI diseases; (4) articles were written in English; and (5) articles included the period of the COVID-19 pandemic, specifically from January 2020 to May 2022. The snowballing method was also used to identify articles that had not appeared in the original search by applying the same inclusion and exclusion criteria. As a result, a total number of *n* = 42 articles remained and used in this report. Figure 1 shows a detailed outline of the selection process.

### Data synthesis

Data were extracted from each full-text article included in this review. The following information was collected: (1) general details, such as author, design of the study, and country; (2) information on the specific GI pathogens, the impact of the COVID-19 pandemic on each pathogen (percentage change in incidence and/or case country compared with pre-COVID-19), per study; and (3) the investigated time period of each study. Data were entered in Microsoft Excel (version 2211).

## Results

The initial dataset comprised 1466 articles, from which 42 articles were selected for inclusion in this review (Fig. 1). Studies predominantly addressed GI viruses, specifically norovirus (*n* = 19) and rotavirus (*n* = 7), and GI bacteria, including *Campylobacter* (*n* = 13), nontyphoidal *Salmonella* (*n* = 15), *Salmonella* Typhi/Paratyphi (*n* = 4), *Shigella* (*n* = 13), STEC (*n* = 7), and *Listeria* (*n* = 5). Four studies offered broader insights into infectious diarrheal diseases, overall GI complaints, or viral GI infections. Six (*n* = 6) out of the 41 studies examined the impact of the pandemic on GI viruses in hospitalized children.

Table 2 provides an overview of investigated GI pathogens per article by region and country, and Table 3 describes their main outcomes, data source, and study period.

**Table 2.** Counties Included in This Review and the Investigated Gastrointestinal Pathogens per Country and Study

Region	Country	N studies/country	References	Gastrointestinal pathogens investigated per study
Pacific	Australia	5	(Xie et al., 2020) (Bruggink, 2022) (Davis et al., 2022) (Bruggink, 2022) (Adegbija et al., 2021)	Rotavirus, *Shigella*, nontyphoidal *Salmonella* Norovirus Nontyphoidal *Salmonella* Norovirus *Campylobacter*, nontyphoidal *Salmonella*, *Shigella*, STEC, rotavirus
North America	United States	8	(Lennon et al., 2020) (Nachamkin et al., 2021) (Ray et al., 2021) (Bulterys et al., 2021) (Collins et al., 2022) (Palmer et al., 2022) (Kambhampati et al., 2022) (Burnett et al., 2022)	Norovirus *Campylobacter*, nontyphoidal *Salmonella*, *Shigella*, norovirus *Campylobacter, Salmonella,* STEC*, Shigella, Listeria* Norovirus, rotavirus*, Campylobacter, Salmonella,* STEC*, Shigella* *Campylobacter,* nontyphoidal *Salmonella,* STEC*, Shigella, Listeria* STEC, norovirus Norovirus Rotavirus
	Canada	1	(Dougherty et al., 2023)	*Salmonella, Shigella, STEC, Listeria*
Western Europe	Germany	5	(Mack et al., 2021) (Eigner et al., 2021) (Ullrich et al., 2021) (Maison et al., 2022) (Terliesner et al., 2022)	Norovirus, rotavirus, *Campylobacter* Norovirus Rotavirus, norovirus, *Shigella,* nontyphoidal *Salmonella, Campylobacter* Norovirus, rotavirus Rotavirus
	England	2	(Hayes et al., 2023) (Love et al., 2022)	Norovirus, *Shigella* Norovirus, nontyphoidal *Salmonella, Campylobacter, Shigella, Listeria,* STEC
	Netherlands	1	(Mughini-Gras et al., 2021)	Nontyphoidal *Salmonella*
Northern Europe	Finland	1	(Kuitunen et al., 2022)	Norovirus, rotavirus
	Denmark	1	(Nielsen et al., 2022)	*Campylobacter,* nontyphoidal *Salmonella*
Central Europe	Poland	1	(Czerwińska and Szenborn, 2020)	Rotavirus, norovirus, adenovirus
	Switzerland	1	(Steffen et al., 2020)	*Campylobacter, Salmonella, Shigella*
Southern Europe	Spain	2	(De Miguel Buckley et al., 2020) (Maldonado-Barrueco et al., 2022)	*Campylobacter,* nontyphoidal *Salmonella* Norovirus, rotavirus
Southeastern Europe–Western Asia	Türkiye	1	(Duman et al., 2022)	Rotavirus
Middle East	Israel	1	(Bassal et al., 2021)	*Shigella,* nontyphoidal *Salmonella*, *Campylobacter*
Southeast Asia	Thailand	1	(Yorsaeng et al., 2022)	Rotavirus, norovirus
East Asia	South Korea	2	(Ahn et al., 2021) (Kim et al., 2022)	Norovirus, rotavirus*,* nontyphoidal *Salmonella, Campylobacter* Nontyphoidal *Salmonella*
	Japan	2	(Hibiya et al., 2022) (Fukuda et al., 2021)	Rotavirus Rotavirus, norovirus
	China	5	(Li et al., 2021) (Chen et al., 2021) (Lu et al., 2021) (Chan, 2022) (Wang et al., 2022)	Rotavirus (Para)typhoidal *Salmonella*, other infectious diarrheal diseases Cases of infectious diarrhea Rotavirus, norovirus Overall gastrointestinal diseases, typhoidal *Salmonella*
	Taiwan	2	(Lin et al., 2021) (Lai et al., 2021)	(Para)typhoidal *Salmonella* (Para)typhoidal *Salmonella, Listeria*, *Shigella*

EIA, enzyme immunoassay; STEC, Shiga toxin–producing *Escherichia coli*.

**Table 3.** Overview of the 41 Mentioned Studies in This Review, the Investigated Gastrointestinal Pathogens, and the Main Outcomes

Study	Data source	Region, country	Main findings per pathogen during investigated periods
(Xie et al., 2020)	Notifiable disease data from the regional Notifiable Diseases Surveillance System	Northern Territory, Australia	During March 15, 2020, to May 15, 2015, compared to March 15–May 15, 2015–2019, the following was observed for monthly notification rate: **rotavirus** decreased by 67.21% (0.80 vs. 2.44), ***Shigella*** by 19.4% (10.17 vs. 12.61), ***Salmonella* nontyphoidal** by 42.4% (13.83 vs. 24)
(Lennon et al., 2020)	National weekly Norovirus Outbreak rates from the Centers for Disease Control and Prevention	United States	The weekly outbreak rates of **norovirus** decreased by 49% during February 6–June 5, 2020, compared to February 6–June 6, 2019 (326 **vs.** 638, *p* < 0.001)
(Mack et al., 2021)	Regional clinical pathology laboratory serving hospitals, general practitioners and outpatient practices	Three federal states, Germany	The mean positivity ratio of **norovirus** was 3- to 20-fold lower (*p* = 0.0032) in pandemic quarters (PQ: 2Q/2020 through 1Q/2021), compared to prepandemic quarters from 2017 (PPQ: 1Q/2017 through 1Q/2020). The mean positivity ratio for **rotavirus** was nonsignificantly lower in PQ (*p* = 0.31) and for *Campylobacter was* nonsignificantly higher in PQ (*p* = 0.91)
(Eigner et al., 2021)	A prospective laboratory-based surveillance study on the occurrence of norovirus-positive tests among hospitalized patients.	Germany	The monthly positivity rate of **norovirus**-positive samples decreased sharply after January 2020, reaching near 0% as of May and continuing around 0% thereafter. The following changes were observed: January 2020 versus 2019 there was a 9% reduction (20% vs. 22%), February 2020 vs. 2019 there was a 36% reduction (16% vs. 25%), from May until July 2020 it reached nearly 0% compared to 11 − 4.3% in 2019, and from August to December 2020 stayed in 2 − 1% compared to 5 − 17% in 2019
(Ahn et al., 2021)	National surveillance data on infectious gastrointestinal diseases	South Korea	During March 2020–February 2021 compared to the average of 2018–2019 the following was observed for the incidence: **norovirus** decreased by 40.2%, **rotavirus** decreased by 31.8%, ***Campylobacter*** increased by107.9% (nonsignificantly) and ***Salmonella* nontyphoidal** decreased by 73.0% (nonsignificantly)
(Steffen et al., 2020)	National surveillance data on notified infectious diseases	Switzerland	During 2020 compared to 2016–2019 the Incidence of ***Campylobacter*** *reduced by* 59.8% (weeks 14–25). The incidence of ***S. Typhi/Paratyphi*** *reduced by* 50.0 % (weeks 16–27), the incidence of ***Salmonella nontyphoidal*** *reduced by* 41.0% (weeks 14–25), and the incidence of ***Shigella*** reduced by 82.4 % (weeks 14–25)
(Nachamkin et al., 2021)	Laboratory data from an academic medical center laboratory	Philadelphia, USA	***Campylobacter*** yearly positivity rates varied between 1.8% and 3.0% pre-COVID-19, dropped to 1.1% or less in March–May 2020, then returned to higher rates the remainder of 2020. ***Salmonella*** average yearly pre-COVID-19 positive rates ranged from 1.3% to 1.5%, dropped in March–May 2020, then returned to historical levels. For ***Shigella***, historical yearly positive rates were 1.0–1.2% pre-COVID-19. Between March and May 2020, two of the 3 months showed rates less than 1.0%, then returned to historical levels. **Norovirus** positivity rates dropped dramatically from a yearly average of 3.9% in 2016–2019 to 0.76% from March 2020 through the end of 2020
(Li et al., 2021)	Outpatient visits, intestinal infection visits, and rotavirus tests from the Children’s Hospital	Hangzhou, China	A 50% reduction of **rotavirus** test positive rate was observed during January–December 2020 compared to the same period of 2019 (7.15% vs. 14.41%)
(De Miguel Buckley et al., 2020)	Epidemiological Surveillance Network from Madrid Autonomous Community	Spain	Between weeks 1–26 2020, the number of ***Campylobacter*** and ***Salmonella* nontyphoidal** cases reduced by 70% (391 vs. 1308) and 75% (111 vs. 462) respectively, compared to 2019
(Lin et al., 2021)	National Infectious Disease Statistics System (TNIDSS).	Taiwan	In 2020, ***Salmonella* typhoidal** cases reduced by 58% compared to the 2011–2019 average (10 vs. 24), and ***Salmonella* paratyphoid** cases saw a 100% reduction (0 vs. 7).
(Bassal et al., 2021b)	Positive stool samples of diarrheal patients from eight sentinel laboratories, reported to the Center for Disease Control	Israel	From March to July 2020, compared to the same period in 2018–2019, relative risk reductions were observed as follows: ***Shigella*** −86.6%, ***Salmonella*** −33.0%, ***Campylobacter*** −30.0%
(Ray et al., 2021)	Foodborne Diseases Active Surveillance Network (Foodnet)	USA	In 2020, notification rate reductions were observed compared to 2017–2019 as follows: ***Campylobacter*** −23%, ***Salmonella*** −22%, **STEC** −37%, ***Shigella*** −41%, ***Listeria*** −27%
(Chen et al., 2021)	National surveillance data on notified infectious diseases	China	The following notification rate reductions were observed in 2020 compared to 2019: ***S. Typhi*/ Paratyphi** −25% (0.52 vs. 0.70), Other infectious diarrheal pathogens −21% (76.33 vs. 96.35)
(Ullrich et al., 2021)	National surveillance data for notifiable infectious diseases	Germany	Case numbers were reduced as follows for the 10–32 weeks of 2020 compared to the same weeks of 2016–2019: **rotavirus** −83.3% (95% CI: −83.9 to −82.7), **norovirus** −78.7% (95% CI: −79.2 to −78.2) ***Shigella*** −82.9% (95% CI: −87.0 to −77.6), ***Salmonella* nontyphoidal** −45.5% (95% CI: −47.4 to −43.4), ***Campylobacter*** −22.2% (95% CI: −23.4 to −21.0)
(Mughini-Gras et al., 2021)	National laboratory surveillance data	Netherlands	During the 4 quarters of 2020, the following significant decrease in ***Salmonella* nontyphoidal** cases was observed, compared with the same quarter in 2016–2019: Q1 = −2%, Q2 = −54%, Q3 = −57%, and Q4 = −47% Additionally, cases reduced by 37% in Q1 of 2021, compared to 2016–2019
(Bruggink, 2022)	Laboratory data from the Victorian Infectious Diseases Reference Laboratory	Victoria, Australia	A 49% reduction in **norovirus** prevalence was detected between January and September 2020, compared to January 2010–September 2019 (31.4% vs. 61.5%)
(Kim et al., 2022)	Multicenter surveillance data on pediatric invasive bacterial infections	South Korea	***Salmonella* nontyphoidal** cumulative incidence reduced by 59% in 2020 compared to 2018–2019 (9.4% vs. 22.8%)
(Davis et al., 2022)	National surveillance data for notifiable infectious diseases	Australia	***Salmonella* nontyphoidal** notifications reduced by 27% in 2020 compared to the 2015–2019 median
(Nielsen et al., 2022)	National Register on Enteric Infections	Denmark	During spring and summer of 2020 (summarized weeks 14–31) the number of ***Campylobacter*** cases reduced by 30%, and ***Salmonella* nontyphoidal** by 52%, compared to the same weeks of 2016–2019
(Maison et al., 2022)	Laboratory data from a pediatric tertiary care university hospital, between Jan 2017–October 2021	Munich, Germany	NPIs had an immense impact on the occurrence of viral respiratory infections during and after the lockdown periods, but for **norovirus** and **rotavirus** the effect was only minimal
(Maldonado-Barrueco et al., 2022)	Retrospective cohort study in children with viral gastroenteritis from a tertiary care hospital	Madrid, Spain	From March 15, 2020, to March 15, 2021, a 69.1% prevalence decrease (4.7% vs. 15.2%) and a 49% relative incidence decrease (1.74 vs. 3.40), was observed for **norovirus** compared to the same period prepandemic (2019–2020). For **rotavirus**, the prevalence and relative incidence decreased by 67% (0.7% vs. 2.1%) and 40% (0.3% vs. 0.5%), respectively
(Collins et al., 2022)	Foodborne Diseases Active Surveillance Network (Foodnet)	USA	A 10% significant decrease (95% CI: −11.4 to 0.9) was observed for ***Salmonella*** incidence in 2021, compared to 2016–2018. Changes in the incidence of the following pathogens were additionally observed, however, nonsignificant: ***Campylobacter***: −5.5% (95% CI: −11.4 to 0.9), **STEC***: +*8.8% (95% CI: −6.8 to 27.0), ***Shigella***: −14.8% (95% CI: −33.8 to 6.0), ***Listeria***: + 4.6% (95% CI: −8.5 to 20.1)
(Love et al., 2022),	National and regional surveillance data on gastrointestinal infections	England	The following decrease was observed for laboratory-confirmed cases in the first 6 months of 2020, compared to the 5-year average of 2015–2019: **norovirus**: −37.8% (5.6% vs. 9.0%), ***Salmonella* nontyphoidal***: −17% (7.9% vs. 9.5%),* **STEC: no change** *(1.2 vs. 1.2)* ***Listeria:* no change** *(0.2% vs. 0.2%).* The bacterial pathogens ***Shigella***, and ***Campylobacter***, showed significant decreases during phase 3 (n/c), remained low during phase 4 but then began to increase during phase 5, following the 5-year average but with a significantly reduced number of cases reported
(Palmer et al., 2022)	Laboratory data from a routinely collected diagnostics database	9 states, USA	An immediate decrease in **norovirus** PCR positivity percentage was observed between beginning March 15, 2020, and March 29, 2020, with variability per state **STEC** positivity percentage was least impacted by social distancing mandates
(Hayes et al., 2023)	Surveillance data from the UK Health Security Agency, the UK Office for National Statistics, and the Royal College of General Practitioners Research and Surveillance Center	England	A substantial drop in **norovirus** cases during the first lockdown (mid-March 2020–June 2021) was described, with less than 10 weekly reported cases compared to a weekly 50–200 cases in 2018. **Norovirus** cases remained low until restrictions were eased in July 2021 and started to increase in the “Living with Covid-19 period” (March–May 2022) but did not reach previous years peaks A similar pattern for ***Shigella*** weekly cases was additionally observed
(Bruggink, 2022)	Laboratory data from the Victorian Infectious Diseases Reference Laboratory	Victoria, Australia	An increase in the number of **norovirus** outbreaks was observed from *n* = 137 in the average 2017–2019 to *n* = 142 between January 2020 and December 2021. During January–December 2020 outbreak numbers were low, equal to *n* = 26
(Lu et al., 2021)	National Notifiable Infectious Disease Reporting Information System (NIDRIS)	Guangzhou, China	A 23% reduction in the number of infectious diarrhea cases was detected in 2020 compared to 2015–2019 (12.065 vs. 15.716): Number of reported cases gradually increased above the average level between September and December 2020
(Chan, 2022)	Routine laboratory syndromic surveillance data	Hong Kong, China	**Rotavirus** positivity decreased abruptly by 70% during February 2020, and remained at a much lower level of 0.1–0.6% through September 2020, compared with a median of 5.4% during the same period in the previous 7 years. In winter 2020–2021, a typical seasonal peak with a positive rate of 10.3% was observed in January 2021, a rate comparable with the median of 14.4% during the previous 7 winter seasons **Norovirus** positive rates decreased sharply by 56% during February 2020 and remained at a much lower level of 0.3–1.5% through September 2020, compared with a median of 6.4% during the same period in the previous 7 years. In winter 2020–2021 a typical seasonal peak with a positive rate of 4.8% was observed in February 2021, highly comparable with the rates of 5.3% and 6.2% in the previous 2 winter seasons
(Adegbija et al., 2021)	Communicable notifiable disease data from the Queensland Notifiable Conditions System (NoCS)	Central Queensland, Australia	From April 1 to September 30, 2020, compared to the previous 5-year average for the same months, the following reductions in disease notifications were observed: **rotavirus**: −91% (3 vs. 35), ***Campylobacter***: *−8% (145 vs. 158),* ***Salmonella* nontyphoidal***: −26% (90 vs. 122).* ***Shigella*** notifications increased by 1% (8 vs. 4), STEC**:** *remained the same*
(Bulterys et al., 2021)	Laboratory data from the Stanford Health Care Clinical Microbiology Laboratory	Northern California, USA	Between March 20, 2020, and September 30, 2020 (after SIP) the following reductions in test positivity ratio were detected, compared to January 1, 2018–March 19, 2020 (before SIP): **norovirus**: −70% (1.16 vs. 5.7), **rotavirus**: −55% (0.55 vs. 1.22), ***Campylobacter****: −22% (*2.01 vs. 2.57), ***Salmonella enterica****: −8%* (1.22 vs. 1.33), **STEC***: −45%* (0.24 vs. 0.44), ***Shigella****: −50% (*0.55 vs. 1.12).
(Hibiya et al., 2022)	Notifiable diseases data from the National Epidemiological Surveillance of Infectious Diseases (NESID)	Japan	In 2020, **rotavirus** notification rate reduced by 94% compared to 2019 (0.52 vs. 9.82)
(Kambhampati et al., 2022)	Norovirus outbreaks reported to the National Outbreak Reporting System by Norovirus Sentinel Testing and Tracking Network	12 states in USA	The number of **norovirus** outbreaks in the 12 states decreased three-fold during the 2020–2021 surveillance years (August 1, 2021–July 31, 2022) and returned to prepandemic levels in 2021–2022 (nearly three times the number reported during the 2020–2021)
(Kuitunen et al., 2022)	A retrospective nationwide register‐based surveillance study using data from the Care Register of Primary Healthcare and the National Infectious Disease Register	Finland	2020 yearly incidence reduced by 70% for norovirus and 64% for rotavirus, compared to 2018–2019. For 2021 the reduction was 46% and 72% for the two viruses, respectively
(Yorsaeng et al., 2022)	Department of Disease Control and King Chulangkorn Memorial Hospital	Bangkok, Thailand	In 2020, prior to the lockdown, the number of **rotavirus** cases rose by over 10 times (+950%) compared to 2019 (158 cases vs. 15 cases). During the lockdown, the number of cases dropped to 6 (6 cases vs. 1 case), and during the easing period (0 cases vs. 3 cases) and the new wave (0 cases vs. 6 cases), no cases were reported After an initial spike in early 2020, the total number of **norovirus cases** in 2020 significantly decreased by 57% compared to 2019 (59 cases vs. 138 cases)
(Duman et al., 2022)	Regional laboratory electronic records of hospitals in Malatya	Malatya, Türkiye	**Rotavirus** monthly median positivity rate reduced by 38% during the pandemic (April 2020–July 2021) compared to prepandemic years (January 2018–March 2020)
(Czerwińska and Szenborn, 2020)	National surveillance data for infectious diseases	Poland	The average number of new cases of viral gastrointestinal infections reduced by 63% in 2020 compared to 2019 (January 1–May 15)
(Lai et al., 2021)	National surveillance data on notifiable infectious diseases	Taiwan	From January to September 2020 the following reduction in the number of cases was observed, compared to 2019: ***S.* paratyphi***:-100%* (0 vs. 7), ***S. typhi****: −65% (*7 vs. 20), ***Listeria****: −23.4%.* ***Shigella*** *cases though, increased by +7.5%* (115 vs. 107)
(Burnett et al., 2022)	Laboratory data reported to the National Respiratory and Enteric Virus Surveillance System (NREVSS)	USA	**Rotavirus** EIA test positivity reduced by 73% (95% CI: 67–79%, *p* < 0.001) during the pandemic period, compared to the vaccine period before the pandemic (2007–2019) Additionally, rotavirus PCR test positivity reduced by 85% (95% CI: 80–87%, *p* < 0.001), compared to the vaccine period of 2012–2019
(Terliesner et al., 2022)	A retrospective study on stool samples from children admitted to the Berlin University Children’s Hospital	Berlin, Germany	**Rotavirus** mean monthly rate of test positivity reduced by 87% during January to May 2020–2021 compared to 2016–2019 (0.29 vs. 2.30). No increase was detected after withdrawal of NPI measures in June 2021, but a slight increase corresponding to the prepandemic seasonality was detected in January 2021
(Wang et al., 2022)	National surveillance data on notifiable infectious diseases	China	In 2020 the following reductions in monthly incidence rates were observed, compared to 2015–2019: **overall GI diseases**: −45.28% (95% CI: −45.41 to −45.15, *p* < 0.001), ***S. typhi***: −35.74% (95% CI: −38.10 to −33.38, *p* < 0.001), **viral GI diseases**: −63.87% (95% CI: −64.03 to −63.72, *p* < 0.001)
(Fukuda et al., 2021)	A multicenter prospective study in hospitalized pediatric patients in 18 hospitals	Hokkaido Prefecture, Japan	Post-COVID-19 (July 2020–February 2021) compared to pre-COVID-19 (July 2019–February 2020) **rotavirus** and **norovirus** number of patients reduced by 2.6% and 27.8%, respectively
(Dougherty et al., 2023)	Weekly counts of laboratory-confirmed cases, obtained from laboratory surveillance data	Canada	In 2020, the following statistically significant changes in the incidence rates were observed compared to the prepandemic 5-year reference period: ***Salmonella***: −39%, ***Shigella***: −67%, ***Escherichia coli* O157**: −46%, **non-O157 *STEC:*** −24%, ***Listeria* monocytogenes**: no significant change

Outcomes are associated with the study design and study period, used per study. We report the percentage change for every pathogen in the given comparison period, and it was calculated by us when it was not mentioned in the individual studies, if possible. Investigated pathogens are highlighted in bold (n/c: noncalculated by referenced study or us).

EIA, enzyme immunoassay; STEC, Shiga toxin–producing *Escherichia coli; SIP, shelter-in-place.*

### GI viruses and observed trends, by world region

#### Observed trends in the pacific

In Australia, during the period of stringent measures (March 15 to May 15, 2020), a 67% reduction in rotavirus incidences was observed in the Northern Territory compared to the corresponding period of the previous 5 years (Xie et al., 2020). Meanwhile, in Central Queensland, Adegbija et al. (2021) reported a 91% reduction in rotavirus notifications from April 1 to September 30, 2020, compared to the same months in the previous 5 years. Examining norovirus outbreaks in Victoria during January–September 2020, Bruggink (2022) observed a 49% decline in outbreaks compared to the same period of the previous decade, although a subsequent increase was noted in 2021.

#### Observed trends in North America

Analyzing outbreak data in the USA, Lennon et al. (2020) reported a 49% decrease in weekly norovirus outbreaks in February–June 2020 compared to the same months in 2019. In Philadelphia, Nachamkin et al. (2021) observed that the percentage of norovirus positivity stool went from 3.9% during 2016–2019 to 1.4% from March through December 2020. In Texas, Palmer et al. (2022) reported an immediate decrease in norovirus PCR positivity percentage in the week that social distancing was mandated, based on laboratory data from nine states. Moreover, Kambhampati et al. (2022) saw that the number of norovirus outbreaks in 12 states decreased three-fold during the 2020–2021 surveillance years and returned to prepandemic levels in 2021–2022. In Northern California, Bulterys et al. (2021) noted a 79% and 55% decrease in positive norovirus and rotavirus laboratory tests, respectively, during March–September 2020, compared to pre-COVID-19 years. Last, Burnett et al. (2022) reported a 73% and 85% decline in rotavirus enzyme immunoassay positivity and PCR positivity, respectively, between 2007–2019 and 2020–2021.

#### Observed trends in Western/Northern Europe

In Germany, Eigner et al. (2021) reported a consistently low monthly positivity rate for norovirus-positive samples from May to December 2020 (nearly 0%), contrasting with rates of 4–11% in May to July 2019. Ullrich et al. (2021) reported substantial decreases in rotavirus (−83%) and norovirus notifications (−79%) between weeks 10–32 in 2020 compared to the same weeks in 2016–2019. Furthermore, 3- to 20-fold lower positivity ratios were reported for norovirus by Mack et al. (2021) based on laboratory data from a large regional laboratory in Western Germany. The ratio for rotavirus was also lower but not statistically significant. Terliesner et al. (2022) observed that the mean monthly rate of children who tested positive for rotavirus in the Berlin University Children’s Hospital decreased by 87% from January to May 2020 compared to the same prepandemic period. They also reported an absence of seasonality, with no increase after withdrawal of NPI measures in June 2021, but a slight increase corresponding to the prepandemic seasonality in January 2021. Between February and November 2020, almost no cases of rotavirus were reported by Maison et al. (2022) at their pediatric clinic. However, after the second lockdown ended in March 2021, a rise in rotavirus cases outside the usual season was observed, comparable to the number of cases in 2019. During 2020, an increase in positive norovirus infections was observed compared to prepandemic years (2017–2019), while during the summer of 2021, these infections were detected slightly more frequently compared to previous years since 2017–2020.

Love et al. (2022) reported a 38% decrease in norovirus laboratory-confirmed cases in 2020 in England, compared to 2015–2019. Hayes et al. (2023) described a substantial drop in norovirus cases during the first lockdown, with less than 10 weekly cases compared to a weekly 50–200 cases in 2018. They remained low until restrictions were eased.

In Finland, Kuitunen et al. (2022) reported a 70% lower incidence of norovirus detections in primary care for gastroenteritis in 2020 and a 46% lower incidence in 2021 compared to 2018–2019 in children aged 0–14 years. A 64% and 72% lower incidence was observed for rotavirus in 2020 and 2021, respectively. The incidence of norovirus remained low throughout 2020 but experienced a rapid increase in March 2021, surpassing the prepandemic levels. Rotavirus cases, however, remained at low levels.

#### Observed trends in Southern/Central Europe

From March 15, 2020, to March 15, 2021, a 69% and 49% decrease in prevalence and relative incidence, respectively, was observed for norovirus in hospitalized children with viral gastroenteritis in Spain, compared to the same period prepandemic Maldonado-Barrueco et al. (2022). For rotavirus, the prevalence and relative incidence decreased by 67% and 40%, respectively.

In Poland, Czerwińska and Szenborn (2020) observed a 63% drop in rotavirus, norovirus, and adenovirus cases from January 1, 2020, to March 15, 2020, compared with corresponding data from 2019.

#### Observed trends in Southeastern Europe—Western Asia

Duman et al. (2022) reported a 35% reduction in the monthly median positivity rate of rotavirus among children admitted to hospitals in Turkey between April 2020 and July 2021 in comparison to January 2018 to March 2020.

#### Observed trends in (South) East Asia

In South Korea, a significant 40% and 32% reduction was observed in the incidence of norovirus and rotavirus, respectively, following the implementation of pandemic measures from March 2020 to February 2021, compared to the same period in 2018–2019 (Ahn et al., 2021).

Hibiya et al. (2022) reported a 94% reduction in norovirus incidence in 2020 in Japan compared to the same period in 2019. In addition, Fukuda et al. (2021) described findings from a multicenter study, where they found a marked decrease in viral gastroenteritis (28% for norovirus and 3% for rotavirus) among hospitalized children during the post-COVID-19 period (July 2020–February 2021) compared to the prepandemic period (July 2019–February 2020).

A significant 5.56-fold increase in rotavirus cases in Thailand was observed by Yorsaeng et al. (2022) during early 2020 compared to 2019, which significantly declined during and after the lockdown in 2020. After an initial spike in early 2020, significantly less norovirus cases were observed compared to the same weeks of 2019, especially during the easing and new wave periods.

In China, Wang et al. (2022) observed that the monthly incidence rate of GI diseases and viral GI diseases decreased by 45% and 64% in 2020, respectively, compared to 2015–2019. Li et al. (2021) reported a 50% reduction in the positivity rate of rotavirus in Children’s Hospital in Hangzhou in 2020 compared to 2019. In the work of Chan (2022), the positivity rates for GI viruses in Hong Kong were lower in early 2020 compared to 2013–2019, most notable for rotavirus with a 70% reduction, and a 56% decrease for norovirus. In January 2021, however, norovirus and rotavirus rates were back at prepandemic levels. In Southern China, Lu et al. (2021) reported a 23% decrease in infectious diarrhea cases from January to August 2020, compared to the same time period in 2015–2019. However, after schools reopened in September, the number of cases was higher than pre-COVID-19.

### GI bacteria and observed trends, by world region

#### Observed trends in the Pacific

In the Northern Territory of Australia, Xie et al. (2020) reported a 42% and 19% reduction from March 15, 2020, to May 15, 2020, in nontyphoidal *Salmonella* and *Shigella* monthly incidence, respectively, compared to the same period in 2019. Davis et al. (2022) described a reduction in nontyphoidal *Salmonella* notifications of 27% in 2020, compared to the previous 5 years. In Central Queensland, Adegbija et al. (2021) reported 26% and 8% less disease notifications for nontyphoidal *Salmonella* and *Campylobacter*, respectively, during the first 6 months of 2020, while the number of STEC notifications remained the same compared with the previous 5 years.

#### Observed trends in North America

Based on national surveillance data, Ray et al. (2021) described a decrease in incidence of foodborne enteric infections in 2021 compared to 2017–2020, caused by *Campylobacter* (−23%), *Salmonella* (−22%), STEC (−37%), *Shigella* (−41%), and *Listeria* (−27%). In 2021, the incidence was only significantly lower for *Salmonella* (−10%), but to a lesser extent (Collins et al., 2022). In Canada during 2020, there was a noted decrease in the number of reported cases for *Salmonella*, *Shigella*, *Escherichia coli* O157, and non-O157 STEC compared to the previous 5-year period, with the number of reported cases for *Listeria monocytogenes* in 2020 remaining consistent with those from the previous 5 years as reported by Dougherty et al. (2023). In Philadelphia, Nachamkin et al. (2021) described a reduction of *Campylobacter*, nontyphoidal *Salmonella,* and *Shigella* in March–May 2020, which then restored to prepandemic levels. In California, a 50% decrease in *Shigella’s* positivity rate was detected by Bulterys et al. (2021) after shelter orders took place in 2020. On the contrary, the rate for *Campylobacter*, *Salmonella*, and STEC infections remained largely unaffected.

#### Observed trends in Western/Northern Europe

In the Netherlands, Mughini-Gras et al. (2021) observed a significant decrease in nontyphoidal *Salmonella* cases based on national surveillance data, which ranged between 55% and 37% depending on the quarters of 2020 and 2021 compared with 2016–2019.

Love et al. (2022) described a decrease in GI laboratory-confirmed cases during weeks 1–31 of 2020 in England. Particularly nontyphoidal *Salmonella*, *Shigella*, and *Campylobacter* showed a substantial decrease, and no decrease was observed for STEC and *Listeria* compared to the previous 5 years. Hayes et al. (2023) also reported a lower number of *Shigella* cases in England during the pandemic from March 2020 to February 2022.

A 22% reduction of nontyphoidal *Salmonella* cases and a 45% reduction of *Campylobacter* cases were reported by Ullrich et al. (2021) in Germany. However, the largest reduction was observed for *Shigella*, with an 83% decrease.

In Denmark, the number of nontyphoidal *Salmonella* and *Campylobacter* cases decreased by 53% and 30%, respectively, as described by Nielsen et al. (2022).

#### Observed trends in Central/Southern Europe

In Switzerland, the number of *Shigella*, nontyphoidal *Salmonella*, and *Campylobacter* cases in 2020 decreased by 82%, 41%, and 59%, respectively, compared with 2016–2019 (Steffen et al., 2020). In addition, the number of typhoid and paratyphoid *Salmonella* cases was reduced by 50%.

The transmission of *Campylobacter* and nontyphoidal *Salmonella* was suppressed during the COVID-19 pandemic in Spain, with a number of infections being reduced by 70% and 75%, respectively, in 2020, compared to 2019 (de Miguel Buckley et al., 2020).

#### Observed trends in East Asia

Lai et al. (2021) demonstrated an overall 23% reduction in the incidence of 8 out of 11 investigated notifiable fecal–oral transmitted infectious diseases in Taiwan from January to September 2020 compared to 2019. Specifically, the number of cases for paratyphoid fever, typhoid fever, and listeriosis were reduced by 100%, 65%, and 23%, respectively, while a slight increase was observed for shigellosis.

Lin et al. (2021) also reported a 58% and 100% decrease in the number of typhoid and paratyphoid *Salmonella* cases in 2020 compared with the past decade.

In South Korea, Ahn et al. (2021) revealed that the *Campylobacter* incidence was either similar or higher in some months of 2020 compared to the previous 5 years, while *Salmonella* rates decreased but not significantly. On the contrary, Kim et al. (2022) showed a 59% reduction in the cumulative incidence among children over 3 months until 18 years of age with an invasive *Salmonella* infection during weeks with restrictive measures.

In China, a statistically significant 25% decrease in typhoid fever and paratyphoid incidence rates was described by Chen et al. (2021) in 2020 compared to 2019, and a 36% reduction in the monthly incidence rates of typhoid fever in 2020 compared to the previous 5-year average by Wang et al. (2022).

#### Observed trends in the Middle East

In Israel, an 86% reduced incidence of *Shigella* was observed in Israel during March–July 2020 compared to 2018–2019, followed by a lower 33% and 30% reduced incidence for *Salmonella* and *Campylobacter* (Bassal et al., 2021).

## Discussion

Studies included in this review showed marked changes in GI pathogen incidence during the COVID-19 pandemic across 18 countries. The incidence of viral GI infections seemed to have decreased more relative to that of bacterial GI infections. This may reflect the relatively more important role of person-to-person transmission in the epidemiology of those viruses, as most NPIs for SARS-CoV-2 were indeed meant to reduce contact between people. Differences in analytical methods, study designs, and periods may explain some of the different outcomes observed among studies. To our knowledge, this is the first review on the effect of the COVID-19 pandemic on the incidence of foodborne pathogens.

### GI viruses and NPIs

The sharp decrease in norovirus and rotavirus infections observed in most studies is often mentioned to be likely related to disruptions in the usual fecal–oral (transmission) route due to constraints in contact between people, especially among children during periods of school closure, among others. The number of hospitalized cases due to GI viruses typically increases during the winter, reaching a peak between January-March in the northern hemisphere (Eigner et al., 2021). However, a steep decrease in the incidence of these viral infections was observed during the winter of 2020 in most studies. As assumed by several authors, a combination of social distancing, remote work and study, increased hand hygiene and cleaning in general, and face masking have been suggested to hinder the transmission of GI viruses too (Adegbija et al., 2021; Ahn et al., 2021; Chan, 2022; Czerwińska and Szenborn, 2020; Duman et al., 2022; Eigner et al., 2021; Hayes et al., 2023; Li et al., 2021; Love et al., 2022; Maldonado-Barrueco et al., 2022; Nachamkin et al., 2021; Ullrich et al., 2021; Wang et al., 2022; Xie et al., 2020; Yorsaeng et al., 2022). Norovirus is also highly contagious through the consumption of contaminated food and water, giving opportunities for frequent outbreaks in institutions and restaurants (Eigner et al., 2021). Similarly, domestic and international border closures prevented the importation of cases from endemic countries, closure of restaurants and other institutions, as well as restricted classroom-based education among children (Bulterys et al., 2021; Eigner et al., 2021; Ullrich et al., 2021; Xie et al., 2020) appeared to have contributed as well. Eigner et al. (2021) also suggested preventing behavioral changes preceding the restriction measures in Germany (i.e., social distancing measures, hand sanitizers) as a justification for the decrease in positivity rates already from February, thus 1-month prior to the implementation of these measures. Specifically for young children, the main suspect of the decreased viral incidence for norovirus and rotavirus is the reduced child-to-child contact as the result of the closing of classrooms and playgrounds, as suggested by Maldonado-Barrueco et al. (2022) in Spain and by Fukuda et al. (2021) in Japan.

With the lifting of control measures, multiple countries reported an increase in the number of rotavirus infections (Kambhampati et al., 2022; Li et al., 2021). The increase in norovirus outbreaks during the first quarter of 2021 in Australia was predominantly associated with childcare settings (Bruggink, 2022). Outbreaks in healthcare settings in Australia (including aged care) were fairly limited at that time, while prior to the COVID-19 pandemic, the majority of outbreaks were attributed to healthcare settings. Bruggink (2022) associated this shift in the outbreak demographics with increased compliance in personal protective equipment by healthcare workers and restriction in visitors, but also with reduced diligence of young children around hand hygiene. Also, other countries reported the return of rotavirus and norovirus activity to pre-COVID-19 levels after an initial decrease, which is suspected to be due to waning immunity that led to an increased susceptible population, while measures were still in place (Chan, 2022; Love et al., 2022).

### GI bacteria and NPIs

Bacterial pathogens faced relatively less significant decreases in incidence during the COVID-19 pandemic. Generally, *Campylobacter* and nontyphoidal *Salmonella* had lower reductions compared to *Shigella* and STEC infections. The highest reduction of shigellosis could be due to the predominant transmission route in some countries, that is, person-to-person, compared to salmonellosis and campylobacteriosis, which are most often foodborne (Bassal et al., 2021; Dougherty et al., 2023; Love et al., 2022). Therefore, most arguments previously suggested for the viruses (e.g., social distancing, increased hygiene) would also apply to *Shigella* (Bassal et al., 2021; Lai et al., 2021). The shift toward preparing food at home due to stay-at-home orders, along with less eating out, fewer social gatherings, improved handwashing, and the implementation of safer food handling practices, likely contributed to the reduction in *Campylobacter* and nontyphoidal *Salmonella* infections (Nachamkin et al., 2021; Ray et al., 2021; Xie et al., 2020). Moreover, the closure of restaurants and food service providers in schools, hotels, and catering businesses may have also contributed to decreased exposure (Davis et al., 2022; Steffen et al., 2020).

Since GI infections can often be acquired abroad while traveling, several studies also highlighted the contribution of entry and travel restrictions (e.g., international or non-essential national traveling) in the reduction of bacterial pathogens (Collins et al., 2022; Lin et al., 2021; Love et al., 2022; Mughini-Gras et al., 2021; Nielsen et al., 2022; Ray et al., 2021).

The return of bacterial GI pathogen incidence to pre-COVID-19 levels in some countries, only a few months after implementing the measures, cannot be fully explained (Nachamkin et al., 2021). A possible reason could lie in their normal yearly variations (Xie et al., 2020) and their typical increasing trends during summertime (Bassal et al., 2021). However, the variety of potential transmission pathways makes it difficult to identify the causative factors (Ahn et al., 2021).

### Additional factors besides NPIs explaining changes in GI pathogens

Although NPIs undoubtedly decreased the number of GI infections, the reasons behind the detected changes are complex and multifactorial. Other factors include changes in healthcare-seeking behavior, as shown by a drop in general practitioner and emergency department visits, as well as less hospital admissions due to diseases other than COVID-19. This was coupled with high pressure on diagnostic services and reduced healthcare capacity, leading to potential under-ascertainment and under-reporting of GI infections (Bassal et al., 2021; Burnett et al., 2022; Collins et al., 2022; Dougherty et al., 2023; Eigner et al., 2021; Lennon et al., 2020; Love et al., 2022; Ullrich et al., 2021). Even though a change in eating habits may have occurred, that is, people ate more often at home, individuals still remained at risk for foodborne infections, supporting the aforementioned hypothesis of under-ascertainment and underreporting (Ullrich et al., 2021).

Most importantly, during public health emergencies, the efficacy of routinely services might be negatively affected. For instance, a reduced ability to properly conduct public health surveillance and outbreak investigation, especially for foodborne outbreaks, was reported in the USA (Kintziger et al., 2021). Because most notification systems depend on infectious disease diagnoses by physicians and laboratories (Ullrich et al., 2021), disruptions in their way of working and patient behaviors might impact the number of cases captured by the systems. Therefore, the extent of the true change in GI infection incidence attributed to NPIs or these other factors cannot be fully discerned.

### Limitations and future research

While our study provides a comprehensive overview of the impact of NPIs on GI infections during the COVID-19 pandemic across various countries, there are some limitations that should be taken into consideration. First, world regions were not equally represented in this review, since no studies were included from Africa and Central and South America. Therefore, findings may not reflect all continents. Second, the reported changes in incidence and other epidemiological metrics were analyzed in the context of the periods during which the NPIs were implemented per study. However, we did not report the specific timing, duration, or strictness of each measure across different regions, which also impacted the reported changes in incidence. Third, the search terms used in this review were chosen to target specific pathogens (e.g., STEC instead of the broader term *E. coli*) to maintain a clear and precise focus on the study’s objectives and to avoid many articles matching the search terms that would not be relevant. However, it may have led to the exclusion of some relevant studies that used broader terms to define, for example, STEC. Fourth, studies that were published after the data extraction in May 2023 may have offered additional insights or revealed different trends that were not captured in this review.

In order to determine the effects of specific NPIs on foodborne disease incidence and herewith insight into the importance of specific pathways, such as the closure of food establishments, future studies could group together countries and/or periods with similar type and strictness of NPIs. Comparison should take into account the differences in public health infrastructure, surveillance systems, and diagnostic practices across different countries and regions. However, because measures such as travel restrictions and closure of food establishments were likely often implemented at the same time, it can be difficult to determine their independent effect on the incidence of foodborne pathogens.

As the world moves to a post-COVID-19 pandemic period, ongoing surveillance of GI pathogens is needed to monitor whether the observed reductions during the pandemic are sustained or whether incidence rates return to prepandemic levels. For example, increased circulation of some foodborne pathogens could occur due to increased population susceptibility and could last several years, as was predicted for norovirus (Lappe et al., 2023).

## Conclusions

A substantial reduction in the incidence of reported GI infections appears to have occurred during the COVID-19 pandemic, with a relatively larger impact on viral vs. bacterial GI pathogens. This is most likely due to the generally higher proportion of viral vs. bacterial infections acquired through person-to-person contact instead of, for example, foodborne transmission, as the implemented NPIs were meant to control SARS-CoV-2, a virus transmitted by person-to-person contact. However, the reasons behind these observed reductions remain difficult to discern at full, as they are likely to be multifactorial in nature. Other factors, such as disruptions in healthcare-seeking behaviors and diagnostic practices, may have also played a significant role in the observed trends. Consequently, the extent of the reduction attributed to NPIs cannot be fully determined. Nonetheless, findings provide insights into which and how potential interventions might also help controlling GI pathogens.
